# The Proteome of Extracellular Vesicles Produced by the Human Gut Bacteria Bacteroides thetaiotaomicron *In Vivo* Is Influenced by Environmental and Host-Derived Factors

**DOI:** 10.1128/aem.00533-22

**Published:** 2022-08-02

**Authors:** Régis Stentz, Emily Jones, Rokas Juodeikis, Udo Wegmann, Maria Guirro, Andrew J. Goldson, Arlaine Brion, Catherine Booth, Padhmanand Sudhakar, Ian R. Brown, Tamás Korcsmáros, Simon R. Carding

**Affiliations:** a Gut Microbes and Health Research Programme, Quadram Institute Bioscience, Norwich, United Kingdom; b School of Chemistry, University East Anglia, Norwich, United Kingdom; c Biochemistry and Biotechnology Department, Nutrigenomics Research Group, Universitat Rovira i Virgili, Tarragona, Spain; d Eurecat, Centre Tecnològic de Catalunya, Centre for Omic Sciences (COS), Joint Unit Universitat Rovira i Virgili-EURECAT, Unique Scientific and Technical Infrastructures (ICTS), Reus, Spain; e Core Science Resources Quadram Institute Bioscience, Norwich, United Kingdom; f Earlham Institute, Norwich, United Kingdom; g Department of Chronic Diseases, Metabolism and Ageing, TARGID, KU Leuven, Leuven, Belgium; h School of Biosciences, University of Kent, Canterbury, United Kingdom; i Norwich Medical School, University East Anglia, Norwich, United Kingdom; Norwegian University of Life Sciences

**Keywords:** bacterial extracellular vesicles, proteome, intestine, microbiota, *Bacteroides thetaiotaomicron*

## Abstract

Bacterial extracellular vesicles (BEVs) released from both Gram-negative and Gram-positive bacteria provide an effective means of communication and trafficking of cell signaling molecules. In the gastrointestinal tract (GIT) BEVs produced by members of the intestinal microbiota can impact host health by mediating microbe-host cell interactions. A major unresolved question, however, is what factors influence the composition of BEV proteins and whether the host influences protein packaging into BEVs and secretion into the GIT. To address this, we have analyzed the proteome of BEVs produced by the major human gut symbiont Bacteroides thetaiotaomicron both *in vitro* and *in vivo* in the murine GIT in order to identify proteins specifically enriched in BEVs produced *in vivo*. We identified 113 proteins enriched in BEVs produced *in vivo,* the majority (62/113) of which accumulated in BEVs in the absence of any changes in their expression by the parental cells. Among these selectively enriched proteins, we identified dipeptidyl peptidases and an asparaginase and confirmed their increased activity in BEVs produced *in vivo*. We also showed that intact BEVs are capable of degrading bile acids via a bile salt hydrolase. Collectively these findings provide additional evidence for the dynamic interplay of host-microbe interactions in the GIT and the existence of an active mechanism to drive and enrich a selected group of proteins for secretion into BEVs in the GIT.

**IMPORTANCE** The gastrointestinal tract (GIT) harbors a complex community of microbes termed the microbiota that plays a role in maintaining the host’s health and wellbeing. How this comes about and the nature of microbe-host cell interactions in the GIT is still unclear. Recently, nanosized vesicles naturally produced by bacterial constituents of the microbiota have been shown to influence responses of different host cells although the molecular basis and identity of vesicle-born bacterial proteins that mediate these interactions is unclear. We show here that bacterial extracellular vesicles (BEVs) produced by the human symbiont Bacteroides thetaiotaomicron in the GIT are enriched in a set of proteins and enzymes, including dipeptidyl peptidases, an asparaginase and a bile salt hydrolase that can influence host cell biosynthetic pathways. Our results provide new insights into the molecular basis of microbiota-host interactions that are central to maintaining GIT homeostasis and health.

## INTRODUCTION

The human gastrointestinal tract (GIT) accommodates a vast and complex community of microbes (the microbiota) that carry out vital functions for human health, including digestion, pathogen exclusion, and immune function (1–3). Increasing our understanding of the basis of this mutualistic relationship and its impact on human health and disease is dependent on defining the pathways and mediators of microbiota-host cross talk. In addition to microbe- and host-derived soluble factors (4, 5) recent evidence suggests BEVs (6) containing various macromolecules can contribute to interactions with other members of the microbial community but also with host cells (7–11).

Nanosized BEVs represent a novel secretion system enabling the dissemination of membrane-encapsulated cellular materials, including proteins, nucleic acids, and metabolites into the extracellular milieu (12, 13) and beyond (10). These include membrane vesicles (MVs) produced by Gram-positive bacteria, and outer membrane vesicles (OMVs) and outer-inner membrane vesicles (OIMVs) (11, 14–16) produced by Gram-negative bacteria. The mammalian GIT contains over 1,000 bacterial species capable of producing BEVs that are implicated in host activities such as digestion and in the development and functioning of the immune system (8, 17, 18). Bacteroides thetaiotaomicron (Bt) is a well-studied Gram-negative anaerobe residing in the lower GIT of human adults. The BEVs it produces are spherical bilayered (50 to 400 nm) vesicles derived from the cell envelope that contain mainly periplasmic content in their lumen (14, 15). Proteomic studies have shown that members of the *Bacteroides* genus, including Bt, use their BEVs as delivery vehicles for the distribution of hydrolases, such as proteases and glycosidases (19), which in the lumen of the GIT aid in the digestion of complex diet- and host-derived molecules (18). In particular Bt BEVs can degrade mucins (20), polysaccharides (17), phytate and inositol polyphosphate derivatives (21) and activate epithelial and immune cells (8, 22–25). By accessing and transmigrating through boundary epithelial cells they can interact with and be acquired by mucosal immune cells and disseminate more widely via the bloodstream (10, 18, 24). Furthering our understanding of BEV biology in general and of their interactions with the host is, however, dependent on first defining the cargo they carry and then identifying interactions of this BEV cargo with host cell signaling and biosynthetic pathways (16, 26).

Here, we have performed a comparative proteomic analysis of Bt BEVs and their parent cells produced both *in vitro* and *in vivo* in the mouse cecum to determine if the host can influence BEV protein composition. We identified a set of 113 selectively enriched proteins in BEVs produced in the mouse GIT that includes enzymes that target proteins integral to host cell biosynthetic pathways.

## RESULTS

### BEV proteomic profile *in vivo*.

**FIG 1:**
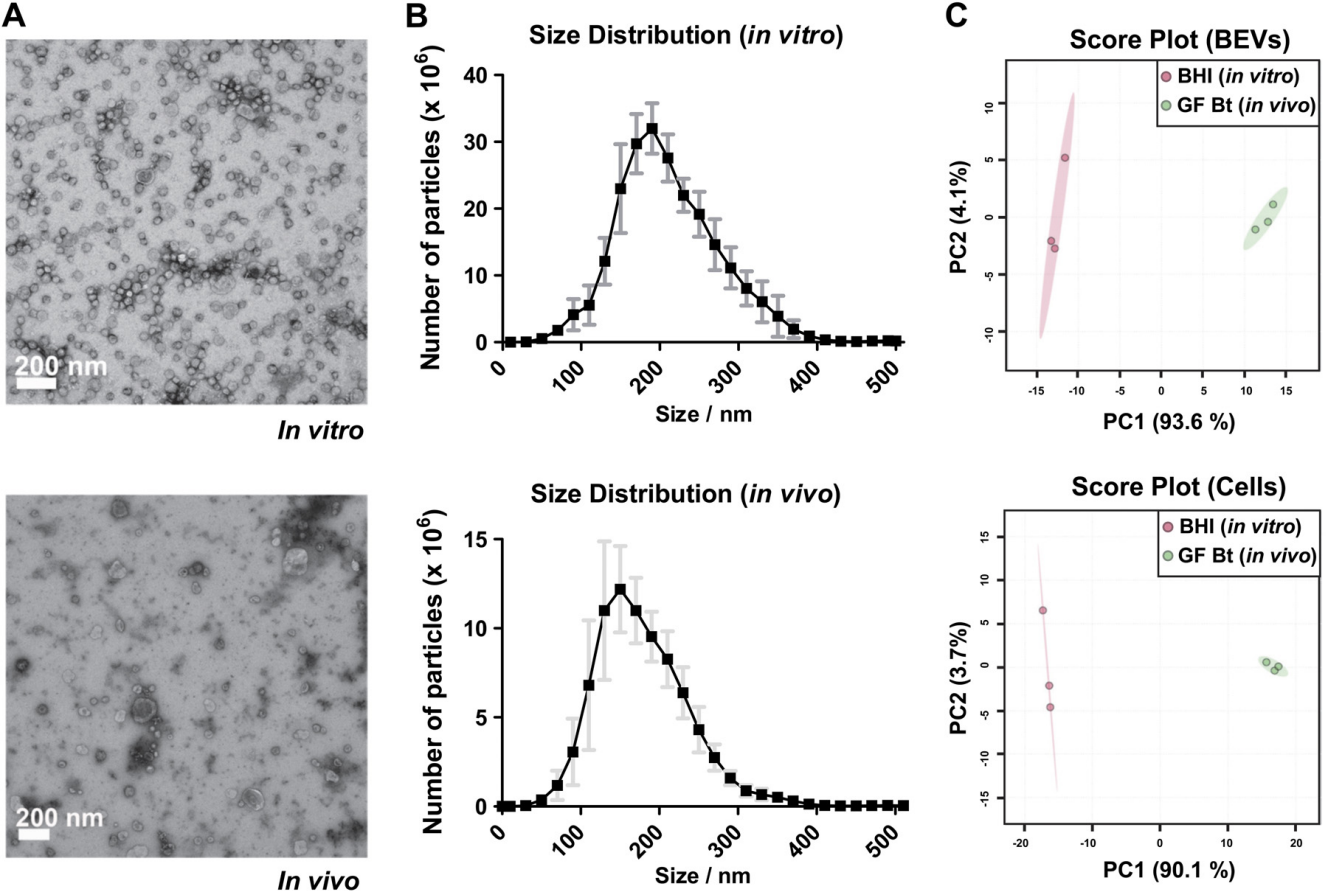
Structure, size, concentration, and protein content of BEVs produced *in vitro* and *in vivo* (A) Transmission electron microscopy generated images of BEVs derived from Bt cells grown in BHI media or from cecal contents of conventionalized germfree mice. (B) Nanoparticle tracking analysis of BEV suspensions. Points (black) represent the mean and the error bars (gray) represent the standard deviation (SD), *n* = 3. (C) Principal Component Analysis performed on normalized abundances of each protein under each condition. X and *y* axis show principal component 1 and principal component 2 explaining 93.6% and 4.1% of the total variance, respectively, for BEV proteins and 90.1% and 3.7%, respectively, for parent cell proteins. Prediction ellipses are such that with probability 0.95 a new observation from the same group will fall inside the ellipse.

Bt produces large amounts of uniform BEV particles that are released from the bacterial cell surface into the external milieu (Fig. S1). To assess the impact of host-related factors on BEV protein composition and identify any BEV proteins enriched *in vivo*, we performed a comparative proteomic analysis of BEVs and their parent cells produced in the complex medium brain heart infusion (BHI) broth or obtained from the cecum of Bt mono-conventionalized germfree mice. We chose to harvest BEVs and cells grown in BHI at early stationary phase to collect BEVs produced throughout exponential growth and minimize contamination with membrane components and cytosolic proteins derived from cell lysis occurring later in the stationary phase. Images obtained by electron microscopy showed that BEVs from BHI and mouse cecum were similar in appearance and structure (Fig. 1A) with the mean size mildly increased in BHI (BHI: 182 ± 16 nm; cecum: 196 ± 18 nm) (Fig. 1B). The yield and recovery of EVs from BHI media (10 ± 2 × 10^11^/500 mL) or cecum (6 ± 1 × 10^11^/mouse) were similar (Fig. 1B). Whereas the extracellular vesicles (EVs) from BHI media consisted primarily of BEVs, the cecal contents of Bt colonized mice contained both host (epithelium)-derived EVs (e.g., exosomes) and BEVs that due to their similar morphologies and appearance (16, 27) made accurate BEV quantification difficult.

Comparative proteomics was carried out on samples of BEVs produced in BHI media and from BEVs and EVs isolated from the cecum of Bt mono-conventionalized germfree mice, and from their parent cells obtained under the same conditions. A total of 2,047 proteins were identified with high confidence (false discovery rate [FDR] < 1%) under both conditions in BEVs and parent cells. To assess consistency of the proteomics data, PCA was performed based on normalized abundances of each protein to obtain an overview for each condition. The PCA scatterplot showed that samples from either BEVs or parent cells formed two distinct clusters depending on whether they were produced *in vitro* or *in vivo* (Fig. 1C) confirming the consistency between biological replicates under each condition. Volcano plots displaying ratios of protein abundances *in vivo* versus *in vitro* (Fig. S2) indicated that for parental cells 372 proteins were more abundant *in vitro*, with 686 more abundant *in vivo* and 989 having no significant change (≥2-fold change, *P* < 0.05, *n* = 3). For BEV proteins 356 were more abundant *in vitro*, 875 more abundant *in vivo*, and 798 having similar abundances in both conditions.

### A set of proteins is selectively secreted in BEVs in the murine GIT.

To investigate whether proteins are selectively enriched in BEVs *in vivo* we first compared the proteome of BEVs harvested from the cecum of Bt mono-conventionalized germfree mice with that of BEVs generated *in vitro* in BHI media. A total of 113 discriminating proteins were identified based upon their abundance being at least 15-fold higher in *in vivo* generated BEVs compared to *in vitro* (Table S1A and B) which are considered “highly” enriched. Next, we compared the levels of expression of the 113 proteins in cecal-derived parental cells with those grown *in vitro* in BHI media to determine if the enrichment of proteins in BEVs *in vivo* was a consequence of their increased production by parental cells (Table S1A and B). We assumed that for a given protein if the enrichment in BEVs *in vivo* occurred independently of protein expression then the *in vivo* versus *in vitro* abundance (ar) of the protein in parental cells would be comparable (i.e., ar < 3-fold change). In contrast, if the enrichment in BEVs *in vivo* was a direct consequence of higher expression in parental cells then its abundance in parent cells *in vivo* would be increased (i.e., ar > 3-fold change) compared to *in vitro* growth conditions.

**FIG 2:**
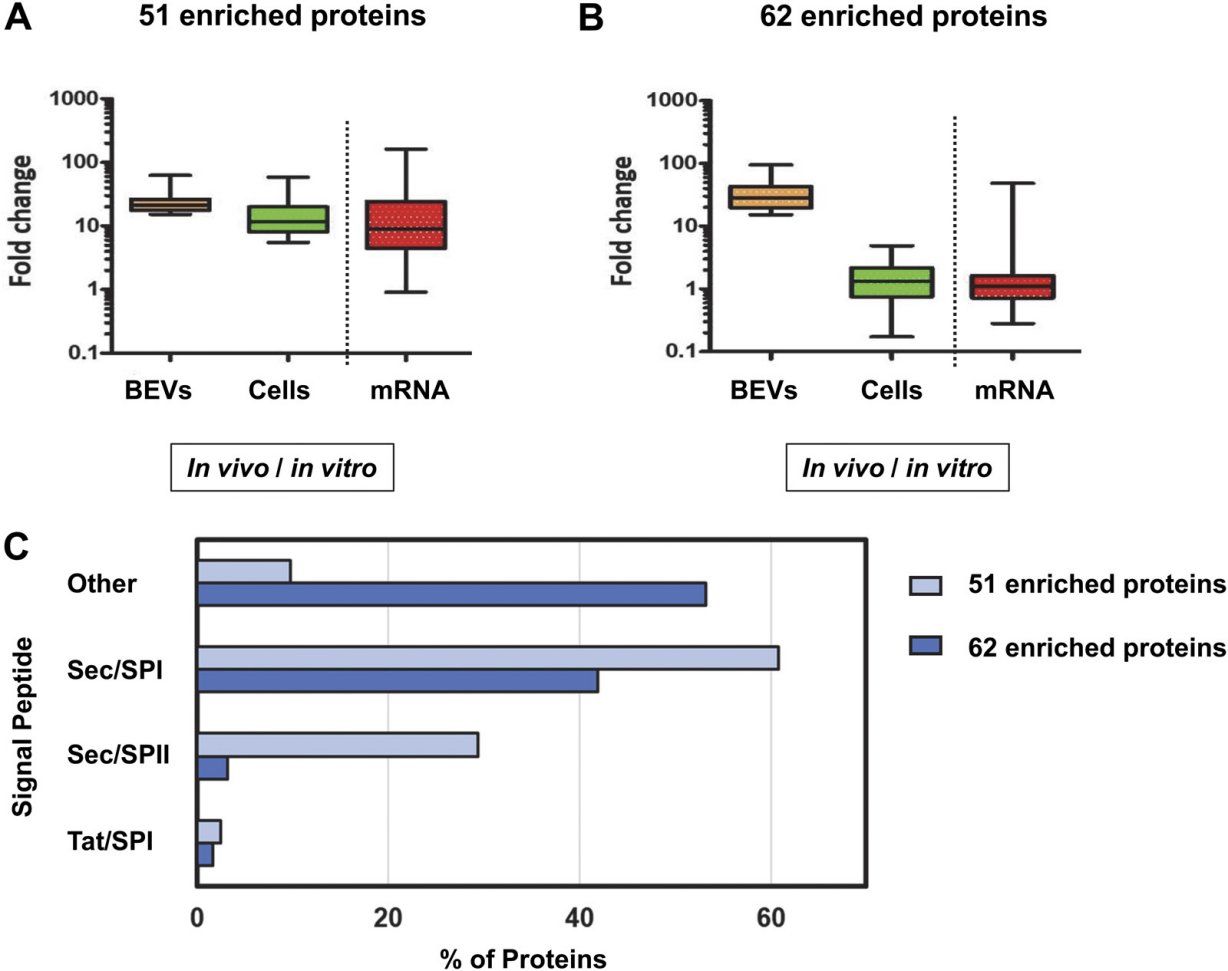
Proteins enriched in BEVs produced in the mouse GI tract. The 113 proteins enriched in BEVs *in vivo* (fold change ≥ 15, Table S1) were divided into two groups based upon comparing their levels in BEVs versus parental cells. Results of the 51 proteins with a greater than 3-fold change in the parent cells *in vivo* (Table S1A) are combined in (A). The 62 proteins with a less than 3-fold increase in abundance in the parent cells *in vivo* (Table S1B) are combined in (B). For these two groups of proteins their expression is compared to that of the mRNA expression level of the corresponding gene in cells grown under similar conditions (28). (C) SignalP-5.0 Server at https://services.healthtech.dtu.dk/service.php?SignalP-5.0 was used to predict the presence of different types of signal peptides present among the two sets of enriched BEV proteins. The set of 51 proteins identified in (A) is represented by light blue bars and the set of 62 enriched proteins is represented by dark blue bars. Sec/SPI are secretory signal peptides transported by the Sec translocon and cleaved by Signal Peptidase I; Sec/SPII are lipoprotein signal peptides transported by the Sec translocon and cleaved by Signal Peptidase II; Tat/SPI are Tat signal peptides transported by the Tat translocon and cleaved by Signal Peptidase I; “Other” are predicted to be nonsecreted proteins and translocated by unknown secretion pathways.

The analysis revealed that 51/113 proteins displayed a 3-fold or higher abundance in parental cells under *in vivo* versus *in vitro* conditions (Table S1A, Fig. 2A) consistent with the increased production in parental cells contributing to their increased abundance in BEVs *in vivo*. In contrast, the analysis revealed that abundances of a majority of proteins (62/113) in parental cells generated *in vivo* or *in vitro* were comparable (Table S1B, Fig. 2B) thereby excluding changes in protein production in parental cells contributing to their increased abundance in BEVs *in vivo.*

To corroborate these findings we compared the mRNA expression levels for each of the 113 proteins enriched in BEVs *in vivo* using data obtained from a global transcriptomics analysis of Bt grown under different *in vitro* and *in vivo* conditions analogous (at https://www.ncbi.nlm.nih.gov/geoprofiles/ Data set GDS1849) to those we have used here (28). Changes in the abundance of the 113 proteins in BEVs generated *in vivo* were closely mirrored by changes in mRNA levels as shown by the fold difference values between protein abundance in the cells and expression of the corresponding gene being significantly correlated (Spearman correlation coefficient r_s_ = 0.81 [*P* < 0.0001]) (Fig. 2A and B, Table S1A and B). These results confirm the selective enrichment of the set of 113 proteins in BEVs generated *in vivo* that occurs either in parallel with (51/113 proteins) or independently of (62/113 proteins) changes in protein production in parental cells.

To predict the presence of known Gram-negative bacterial signal peptides among the 113 proteins enriched in BEVs *in vivo*, we used the SignalP-5.0 Server software program (Table S1). Most of the 51 proteins (46/51) whose abundance values were 15-fold or higher in BEVs *in vivo* versus *in vitro* were predicted to be transported via the bacterial Sec-dependent protein secretion pathway (Fig. 2C). In contrast, 34/62 (54%) of the proteins displaying a 3-fold or less increase in abundance in parent cells *in vivo* were not predicted (labeled other, Fig. 2C) to be secreted through known bacterial secretion systems and are likely to be derived from the cell cytoplasm.

We next aimed to confirm the *in vivo* enrichment of Bt proteins in BEVs by assessing levels of the biological activity of three proteins/enzymes enriched in *in vivo* produced BEVs that can contribute to host biosynthetic pathways: a bile salt hydrolase, dipeptidyl peptidase, and an asparaginase.

### BEV contain a functional bile salt hydrolase selectively increased in BEVs *in vivo*.

The bile salt hydrolase (BSH) BT_2086 was prominent among the 51 proteins enriched in both BEVs and parent cells *in vivo* (Table S1A). BSHs produced by gut commensal bacteria catalyze the hydrolysis of bile salts conjugated with the amino acids taurine or glycine residues and release free bile acids such as cholic acid (CA), glycine, and taurine (29).

**FIG 3:**
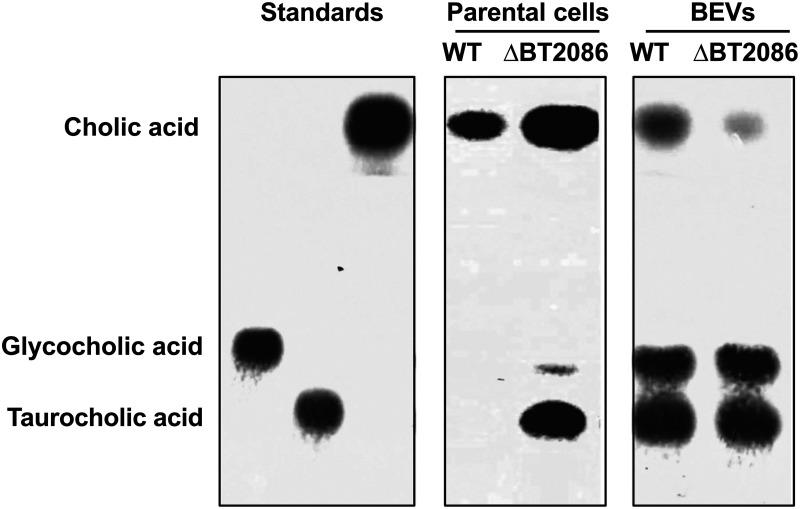
Bile salt hydrolase activity in Bt and BEVs. Thin layer chromatography was used to identify BSH activity and substrate specificity of the BSH encoded by *BT_2086* present in Bt cells and BEVs obtained after growth in BHI media. Cholic acid (CA), taurocholic acid (TCA) and glycocholic acid (GCA) standards were incubated with whole cells or BEVs from wild type (WT) or a Bt BSH1 deletion mutant (*ΔBT_2086*) for 24h at 37°C after which supernatants were spotted onto a silica gel plates. The plate was inserted into a TLC chamber, run for 40 min, and stained with phosphomolybdic acid. The data shown is representative of two experiments.

To confirm that BT_2086 is present and active in BEVs, we first showed that Bt cells grown in BHI *in vitro* degraded both glyco- and tauro-conjugated bile acids (GCA and TCA, respectively) (Fig. 3). The specificity of the BSH encoded by *BT_2086* was established by generating a Bt mutant lacking *BT_2086* (*ΔBT2086*). The mutant was unable to degrade TCA, whereas hydrolysis of GCA was only slightly affected, reflecting the activity of a second predicted BSH, BT_1259 (30). In the case of BEVs, levels of BSH activity and CA production were lower than that of parent cells as reflected in higher residual levels of GCA and TCA after incubation with BEVs (Fig. 3). Importantly, ΔBT2086-generated BEVs produced strikingly less CA compared to BEVs from wild-type Bt (Fig. 3), consistent with Bt BEVs containing a BSH able to deconjugate tauro-conjugated bile salts.

### Enrichment of dipeptidyl peptidase-4 activity in BEVs produced *in vivo*.

The dipeptidyl-peptidase (DPP-4)-like protein predicted to be secreted and encoded by *BT_1314* (DPP-6) was enriched in BEVs *in vivo* (Table 1 and S1B) as well as the DPP-4 enzymes BT_4193 and BT_3254 (Table 1 and S1C). Human DPP-4 or CD26 truncates proteins containing the amino acid proline or alanine in the second position of the N terminus, including the appetite hormones GLP-1 and GIP and neuropeptide substance P, suggesting that DPP-4-like activity encoded by the intestinal microbiome may constitute a novel mechanism to modulate protein digestion and host metabolism (31). We investigated therefore whether intact BEVs could hydrolyze the DPP-4 substrate Ala-Pro-pNa (32).

**TABLE 1:** Dipeptidyl peptidases (DPP) identified in BEV proteome

Accession	Gene ID	Description	Coverage	Number PSMs	Abundance^*a*^ ratio: BEVs	Abundance^*a*^ ratio: cells	Gene expression^*b*^	Prediction^*c*^
Q8A860	BT_1314	DPP4-like, DPP6	29.57	17	21.19	0.73	0.74	SP(Sec/SPI)
Q8A028	BT_4193	DPP4	38.45	61	9.87	2.00	0.86	SP(Sec/SPI)
Q8A2Q1	BT_3254	DPP4	50.68	95	5.98	1.01	0.58	SP(Sec/SPI)

a *In vivo* versus *in vitro*.

b Derived from Sonnenburg et al. (5), gene expression in Bt in mono-conventionalized mice versus Bt grown to early stationary phase in rich medium.

c Signal peptide (SP) prediction using the SignalP-5.0 Server software program. SPI, signal peptidase I.

**FIG 4:**
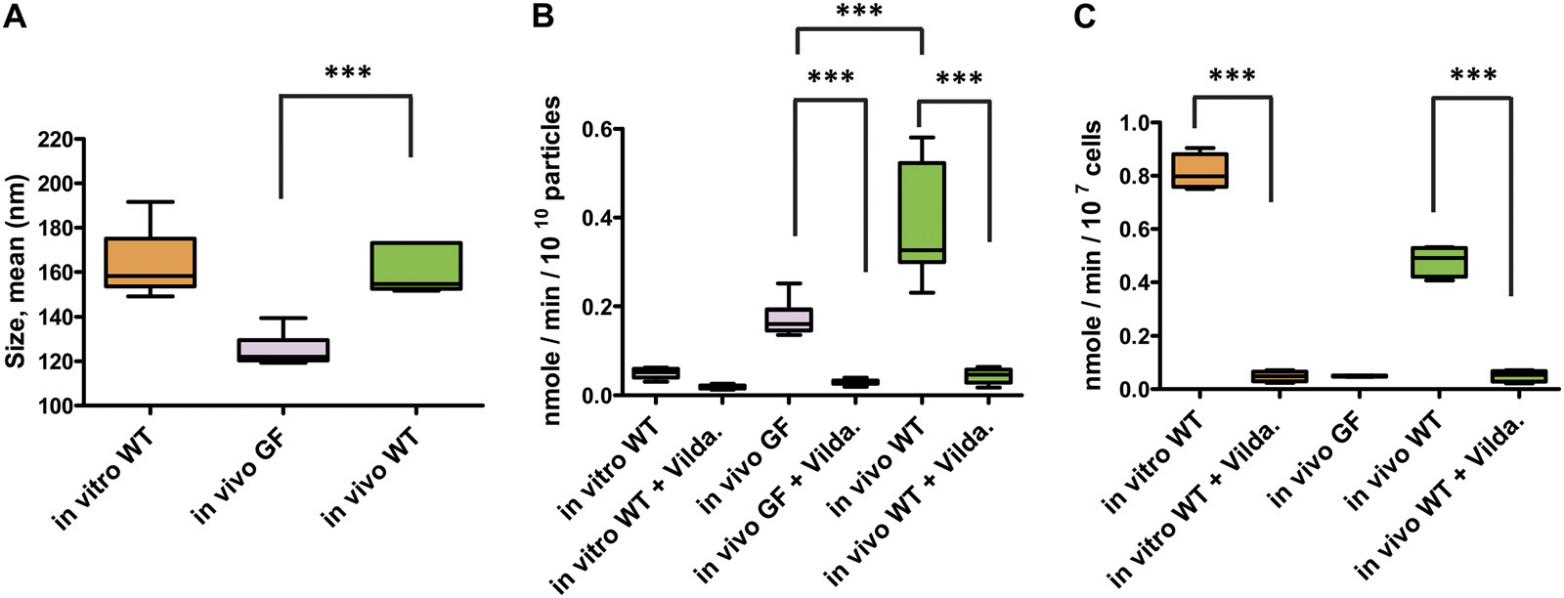
Enrichment of dipeptidyl peptidase activity in BEVs *in vivo.* (A) Nanoparticle tracking analysis was used to determine the size of extracellular vesicles produced by Bt grown *in vitro* or produced in the ceca of conventionalized germfree mice. Following size exclusion chromatography (SEC), fractions F1-F6 from 4 biological replicates were analyzed. The graph is representative of particle sizes measured in the 4 replicates. (B) DPP activity measured in SEC fractionated particles obtained under different conditions (*n* = 4 each). (C) DPP activity measured in protein cell extracts from Bt cells obtained under different conditions (*n* = 4 each). Activities were normalized according to CFU measured for each sample before extraction. *In vitro* WT: vesicles or cells from WT Bt strain grown in culture; *in vivo* GF: particles from germfree mice; *in vivo* WT: particles or cells from germfree mice conventionalized with WT Bt; + Vilda.: particles or cells for which 0.33 μM vildagliptin was added to the reaction mixture. *** = *P* ≤ 0.001.

After extraction and purification, the average size of BEVs produced in BHI *in vitro* by Bt (163 ± 15 nm) was similar to the size of BEVs and EVs produced *in vivo* by Bt (160 ± 10 nm) and significantly larger than EVs produced in the cecum of germfree mice (125 ± 7 nm) (Fig. 4A and S3A).

Consistent with the proteomic data (Table 1), DPP activity measured in BEVs produced *in vitro* was significantly lower than that measured in EVs produced *in vivo* (Fig. 4B). The specificity of the measured activity was assessed using the selective DPP-4 inhibitor vildagliptin (31). In the presence of vildagliptin, 80 to 90% of the activity was reduced in EVs from germfree mice and from Bt conventionalized mice, respectively (Fig. 4B), indicating that most of the measured activity was attributable to DPP-4 in both cases. The activity measured in EVs from mono-conventionalized mice was 2.3 times higher than that of EVs isolated from the cecum of germfree mice (*P* = 7.73 × 10^−11^). This result suggests that around 43% of the DPP-4 activity measured in EVs from Bt conventionalized mice is due to EVs released by the mouse intestinal epithelium, although the contexts are different.

Furthermore, despite there being no detectable DPP-4 activity in BEVs from BHI media consistent with our proteomics data, the enzyme levels in parent cells grown in BHI were increased 1.65 times compared to levels in cells obtained from Bt conventionalized mice (Fig. 4C) reflecting that seen in protein abundances in the proteomics analysis (Table 1 and S1B). Very little DPP-4 activity was detected in fecal pellets from control nonmanipulated germfree mice and in cells treated with vildagliptin (Fig. 4C).

### Enrichment of asparaginase activity in BEVs *in vivo*.

Proteomics data revealed that the secreted type II l-asparaginase encoded by *BT_2757* was 16 times enriched in BEVs *in vivo* (Table S1B). l-asparaginase catalyzes the conversion of l-asparagine to l-aspartate. Therefore, we investigated whether intact BEVs could hydrolyze l-asparagine and if so, whether BEVs produced *in vivo* degraded this substrate more efficiently than BEVs produced in BHI *in vitro*. To determine if asparaginase activity originated from BT_2757 a deletion mutant was used as a control strain.

**FIG 5:**
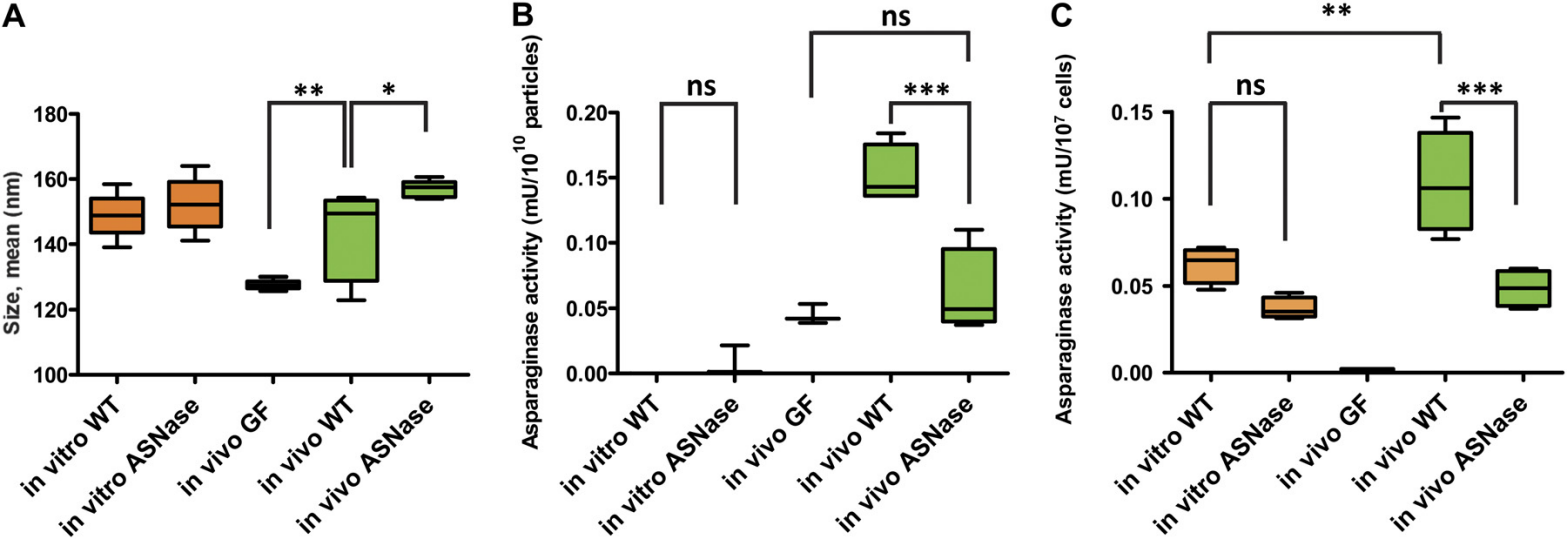
Enrichment of asparaginase activity in BEVs *in vivo.* (A) Nanoparticle tracking was used to determine the mean size of the extracellular vesicle population released in the cecal lumen of germfree mice conventionalized with Bt WT (*in vivo* WT) and *ΔBT_2757* (*in vivo* ASNase) strains or produced in the cecum of nonmanipulated germfree mice (GF). Following size exclusion chromatography (SEC) fractions F1-F6 from 4 biological replicates were analyzed. The graph is representative of particle sizes measured in all replicates. (B) Asparaginase activity measured in combined SEC fractions (F1-F6) of particles extracted under different conditions; *n* = 4 for activity measured in particles extracted from mouse cecum of conventionalized germfree mice, *n* = 3 for vesicles obtained *in vitro* and for EVs obtained from germfree mice. (C) Asparaginase activity measured in protein cell extracts from Bt cells extracted under different conditions, *n* = 4. Activities were normalized according to CFU measured for each sample before extraction. *In vitro* WT = vesicles from WT Bt strain grown in BHI media; *In vitro* ASNase = vesicles or cells from Bt strain *BT_2757* deletion mutant grown in BHI media; *in vivo* GF = particles from nonmanipulated germfree mice; *in vivo* WT = particles or cells from germfree mice conventionalized with WT Bt; *in vivo* ASNase = particles or cells from germfree mice conventionalized with Bt *ΔBT_2757* strain. Ns = not significant, * = *P* ≤ 0.05; ** = *P* ≤ 0.01; *** = *P* ≤ 0.001.

The particle concentration of BEVs and EVs isolated *in vivo* was 1.5-fold higher for mice colonized with the asparaginase (ASNase) mutant (Fig. S3B, fractions F1-F6 combined). The average size of the EVs was significantly larger (*P* = 0.05) for particles obtained from the ASNase mutant (Fig. 5A and S3B) with a size; 143 ± 13 nm for mice conventionalized with wild-type Bt compared to 157 ± 2.5 nm for mice conventionalized with the Bt ASNase mutant. EVs from germfree mice had an average size of 127.6 ± 1.5 nm.

In terms of asparaginase activity, no significant difference was observed between BEVs produced by wild-type Bt and the ASNase Bt mutant in BHI media *in vitro* with both activities being barely detectable (Fig. 5B). A 2.5-fold increase (*P* = 0.0004) was measured for EVs isolated from the cecum of mice conventionalized with Bt compared to EVs isolated from mice conventionalized with the ASNase Bt mutant. Together with the very low activity measured for BEVs produced *in vitro*, it reflects the increased abundance observed in proteomic analyses (Table S1B). The asparaginase activity measured in EVs from germfree mice represented 30% of the activity measured in EVs from wild-type Bt conventionalized mice which may lead to an underestimation of the activity due to BT_2757. A second ASNase was also detected in BEVs *in vivo* (BT_0526, Table S1C and S2) which may also contribute to the activity measured in BEVs.

In comparing asparaginase activity of parental cells, a nonsignificant (*P* = 0.16), 1.6-fold higher level of activity was detected in wild-type versus ASNase mutant cells in BHI media *in vitro* (Fig. 5C) with a significant (*P* = 0.0003) 2.2-fold difference seen in EVs recovered from germfree mice conventionalized with wild-type Bt versus the ASNase mutant. The activity measured in the wild-type Bt was significantly higher *in vivo* (*P* = 0.0023) with a 1.8-fold difference. These differences were comparable to those seen in the proteomics analyses. No asparaginase activity was detectable in fecal pellets of nonmanipulated germfree mice.

## DISCUSSION

Our study provides new insights into microbe-host interactions in the mammalian GIT and how BEVs can contribute to this cross talk. Using the human commensal gut bacterium Bt as a model system, we have identified a set of proteins selectively enriched in BEVs produced in the mouse GIT that include enzymes of potential significance to host physiology.

From previous work on bacterial pathogens, it is known that bacterial proteins, including virulence factors are selectively enriched in BEVs, consistent with vesiculation being a coordinated rather than passive process (8, 11, 33, 34). Virulence factors enriched in BEVs include gingipain proteases produced by the human oral pathogen Porphyromonas gingivalis, or the virulence factors VacA, urease and CagA produced by the gastric pathogen Helicobacter pylori, whereas other abundant cellular proteins not contributing to infection are excluded from BEVs (35, 36). By comparison less is known of the functional potential of BEVs produced by commensal bacteria. Prior studies of the BEV proteome of *Bacteroides* species identified acidic lipoproteins with hydrolytic and carbohydrate-binding activities as being enriched in BEVs (19, 37). Our analysis of BEV proteins under different culture conditions highlights the ability of Bt to respond to environmental cues by changing the profile of proteins it produces and packages as BEV cargo. A similar phenomenon has been described in Campylobacter jejuni, which, although considered to be a commensal bacterium in avian hosts, is pathogenic and causes bacterial gastroenteritis in humans (38). Proteomic analysis of C. jejuni BEVs identified numerous proteins with differential abundance under culture conditions reflecting the different body temperatures of the two hosts, with significantly larger amounts of virulence proteins associated with BEVs from cultures at 37°C culture compared to BEVs produced at 42°C (39).

BEVs of Gram-negative bacteria predominantly comprise OMVs containing secreted periplasmic proteins, outer membrane proteins, and lipids, whereas OIMVs additionally contain cytoplasmic proteins, nucleic acids, and inner membrane components (14, 15, 40). It is now clear that BEV preparations are not homogenous and contain different types of vesicles with distinct compositions (11). The presence of OIMVs in addition to OMVs in our BEV preparations would explain the presence of cytoplasmic proteins in BEV proteomes in both *in vitro* cultures and in the mouse intestine. The determination of the precise mechanisms by which each of these proteins and other types of cargo are selectively packaged in vesicles, although challenging, remains an area of active investigation. The mechanism is likely to involve unique processes as a substantial proportion of proteins highly enriched in BEVs are predicted to be secreted via unknown bacterial secretion systems. Another challenge in defining BEV function *in vivo* is assigning protein and enzyme activity to a specific type of EV and distinguishing BEVs from morphologically similar mammalian-derived EVs. The use of more effective separation techniques in the future will aid in identifying the origin of enzymes and other functional cargo in complex mixtures of EVs and in particular those obtained *in vivo*.

A BLASTP search revealed that the three DPP enzymes BT_1314 (DPP-4-like, DPP-6), BT_4193 (DPP-4), BT_3254 (DPP-4), and the enzymes BT_2086 (BSH) and BT_2757 (ASNase) shown here to be present and active in BEVs produced in the GIT of animals are widely distributed among *Bacteroides* species. Assuming that these proteins are present and active in BEVs produced and released by most, if not all of these species that form one of the most represented genera of the healthy human gut microbiota (41), we predict that large numbers of BEVs containing these enzymes are produced in the lumen of the lower GIT.

The dipeptidyl-peptidases encoded by *BT_1314*, *BT_4193,* and *BT_3254* are enriched in BEVs *in vivo* and have the potential to influence host physiology via their effect on protein and glycan (e.g., gluten) digestion, signal transduction and apoptosis (31). Based upon their ability to cleave and inactivate various signaling molecules important in metabolism (i.e., incretins), the immune system (i.e., growth factors and cytokines), and CNS (i.e., neuropeptides) (42–44), it is tempting to speculate that based upon the ability of Bt BEVs to access the systemic circulation (10, 45), BEVs can impact on various aspects of host physiology and behavior, a possibility that awaits confirmation from further studies.

The type II l-asparaginase encoded by *BT_2757* was also selectively enriched in BEVs *in vivo*. Asparaginase activity is required to deamidate asparagine to aspartate, an essential amino acid for proliferating mammalian cells (e.g., cancer cells) and as a neurotransmitter (46, 47). The human asparaginase enzyme (ASPG) exhibits a relatively low affinity for l-asparagine while bacterial enzymes, which are commonly used as anticancer drugs, have a higher affinity for the substrate (48). Indeed, E. coli-derived asparaginase is used in food manufacturing to reduce levels of the human carcinogen acrylamide (49) and clinically to treat leukemia and lymphoma patients (50). The uptake of aspartate generated from asparaginase cleavage of asparagine is inefficient in most mammalian cells (46). It can therefore be envisaged that following internalization of Bt BEVs into the cytosol of mammalian cells such as intestinal epithelial cells (10), BEVs could supply cells with asparaginase activity and address a shortage of aspartate to aid host cell metabolism.

Our findings demonstrating that Bt BEVs produced in the mouse GIT contain biologically active *BT_2086*-encoded bile salt hydrolase is of potential significance for host physiology. Primary or liver-derived BAs in mammals are mostly conjugated with taurine or glycine and undergo deconjugation by microbial bile salt hydrolases in the small intestine. Although the majority of BAs are transported back into the liver via enterohepatic circulation, small quantities of primary BAs also reach the colon, where certain gut bacteria transform them into secondary BAs by deconjugation, oxidation/epimerization, (7-α-) dehydroxylation and esterification (51). Decreased bile acid deconjugation correlates with inflammatory bowel disorders, including ulcerative colitis, Crohn’s disease, and irritable bowel syndrome (51). To illustrate the importance of BSH in Bt, Yao and colleagues (52) have shown that Bt BSH mutant (Δ*BT_2086*)-colonized mice displayed altered metabolism, including reduced weight gain and respiratory exchange ratios, as well as transcriptional changes in metabolic, circadian rhythm, and immune pathways in the gut and liver. Secondary BAs also exhibit antimicrobial activity conferring them with a key role in shaping the composition of gut bacterial communities (53).

Among the other proteins enriched in BEVs *in vivo*, 22 gene products belonging to the Bt polysaccharide utilization unit loci (PUL) family (54) were identified. This large repertoire of proteins is dedicated to the utilization of diverse plant polysaccharides and host glycans. The BEV PUL proteins included 12 SusC homologs encoding TonB-dependent transporters and 3 SusC-associated substrate-binding SusD lipoproteins. The PUL that includes the largest number of enriched proteins required for the acquisition and degradation of a specific glycan was PUL7 (SusD homolog BT_0363, putative arabinan endo-1,5-alpha-l-arabinosidase BT_0360, SusC homolog BT_0364, SusC homolog BT_0362, Alpha-l-arabinofuranosidase BT_0348), which is part of the 51-protein group. In the PULDB database PUL7 is predicted to be involved in the degradation of “host/residual dietary glycans (unknown type) – arabinan” (55, 56).

Eight galactosidases were also present among the 113 proteins with increased abundance in BEVs *in vivo* (including five β-galactosidases) despite the absence of their substrates (i.e., lactose and derivatives) in the mouse chow suggesting these galactosidases enriched in BEVs *in vivo* may target degradation of host glycoproteins and mucins (57, 58).

Mono-conventionalized germfree mice offer an excellent tool to study the relationship between a single microorganism and its mammalian host. However, it is important to note that the introduced microorganism can reach a higher density and may also establish in different sites within the gut, than would otherwise be the case in the presence of a normal murine microbiota. BEV production and composition may also differ depending on which of these contexts the bacterium is in. In addition, although the mouse offers a number of advantages as an animal model, it will not fully recapitulate human systems and care must be taken in drawing comparisons between mice and humans (59). Germfree mice do however represent a unique system in which to investigate the interaction of a single bacterium with the host, providing the means of developing hypotheses that can be tested further in more complex multibacterial-host systems.

In summary our findings provide evidence for the selective enrichment of proteins in Bt-generated BEVs *in vivo* in the mouse GIT that includes enzymes capable of influencing host cell function and metabolism. Further investigations to evaluate the impact of selected candidates such as BSH, DPP-4-like dipeptidyl-peptidase, or asparaginase on host physiology will help further define determinants of BEV-host interactions that play key roles in the maintenance of intestinal homeostasis and health of the host.

## MATERIALS AND METHODS

### BEV and parent cell preparation from *in vitro* cultures.

The bacterium Bt VPI-5482 was grown anaerobically at 37°C with agitation using a magnetic stirrer in 500 mL of Brain Heart Infusion (BHI) medium (Oxoid/Thermo Fisher, Basingstoke, UK) supplemented with 0.5 mg/L hemin. BHI (three independent cultures) was inoculated with an overnight culture of Bt at an initial OD_600_ of 0.05. After 5 h of growth (OD approximately 2.5, early stationary phase), 10 mL of the culture was collected and centrifuged at 5500 g for 15 min at 4°C, the cell pellets were rinsed twice with 10 mL of PBS, the supernatant removed using a vacuum pump and the pellets snap-frozen in liquid nitrogen and stored at −80°C prior to extraction. For BEV preparations, the remaining bacterial cultures were centrifuged at 5500 g for 45 min at 4°C and the supernatants were filtered through polyethersulfone (PES) membranes (0.22 μm pore-size) (Sartorius) to remove debris and cells. The sterility of the vesicle-containing filtrates was confirmed by plating onto BHI supplemented with 10 mg/L hemin (BHIH) agar. BEVs in the 500 mL filtrates were concentrated by crossflow ultrafiltration (100 kDa MWCO, Vivaflow 50R, Sartorius) to 2 mL, diluted by addition of 500 mL phosphate-buffered saline (PBS), pH 7.4, and the suspensions were concentrated again by crossflow filtration and filter-sterilized through a 0.22 μm PES membrane (Sartorius). Following crossflow ultrafiltration, further purification of BEVs was performed by fractionation of the suspension by size exclusion chromatography using a CL2-B Sepharose (Sigma-Aldrich) (120 cm x 1 cm column) in PBS buffer (24) onto which 1 mL of BEV suspension was loaded. The absorbance of the fractions was measured at 280 nm and the first fractions corresponding to the first absorbance peak were pooled and concentrated to 5 mL with a Vivaspin 20 centrifugal concentrator (100 kDa molecular weight cutoff, Sartorius) and filtered through a 0.22 μm PES membrane (Sartorius). Vesicle concentration was determined by Nanoparticle Tracking Analysis (NTA). The volume of the retentate was adjusted to 8.9 mL and the BEV suspension centrifuged (150,000 g at 4°C or 2 h) in a Ti70 rotor (Beckman Instruments). After centrifugation, the supernatant was removed using a vacuum pump and the vesicle pellets snap-frozen in liquid nitrogen and stored at −80°C prior to extraction.

### BEV and bacterial cell isolation from the mouse cecum.

Three germfree C57BL/6 mice (males,12 to 14 weeks old) maintained in sterile flexible film isolators housed in the QIB Germfree Animal Facility within the Disease Modeling Unit at the University of East Anglia were orally gavaged with 10^8^ CFU Bt in 100 μL PBS. Mice had unrestricted access to chow (Rat and Mouse n°3 breeding, Special Diet Services) and water for 3 days. The study was reviewed and approved by the Animal Welfare and Ethical Review Body (AWERB, University of East Anglia, Norwich, UK) and was conducted within the provisions of the Animals (Scientific Procedures) act 1986. Post mortem (3 days), the cecal contents from the three mice were collected separately and homogenized in PBS (10% wt/vol). Homogenates were centrifuged for 5 min at 1000 g and the supernatant collected. A 100 μL aliquot was removed to enumerate bacteria on BHIH agar (= 12 ± 3 × 10^10^ CFU/g colon content). The supernatants were then centrifuged at 5,500 g, 4°C for 15 min. The cell pellets were rinsed twice with 30 mL PBS and snap-frozen in liquid nitrogen and stored at −80°C prior to extraction. The supernatants were filtered through polyethersulfone (PES) membranes (0.22 μm pore-size) (Sartorius). The sterility of the vesicle (BEV and EV)-containing-filtrate was confirmed by plating onto BHIH agar. Vesicle suspensions were concentrated and the vesicles collected as described above.

### Nanoparticle analysis.

For BEV preparations used in proteomic studies, size distribution of vesicles was performed on 1 mL of BEV suspensions diluted 100-fold with PBS. Videos were generated using a Nanosight nanoparticle instrument (NanoSight Ltd., Malvern Panalytical, USA) to count BEV numbers in BEV samples. A 1-min AVI file was recorded and analyzed using NTA (Version 2.3 Build 0011 RC, Nanosight) software to calculate size distributions and vesicle concentrations using the following settings: Calibration: 166 nm/pixel; Blur auto: Detection threshold: 10, Minimum track length: auto, Temperature: 21.9°C, Viscosity: 0.96 cP. The accuracy of the measurement was confirmed using 100 nm silver nanoparticles (Sigma-Aldrich). For BEV preparations used in *in vitro* assays, the size and concentration of the isolated Bt BEVs was determined using a ZetaView PMX-220 TWIN instrument according to manufacturer’s instructions (Particle Metrix GmbH, Germany). Aliquots of BEV suspensions were diluted 1,000- to 20,000-fold in particle-free PBS or water for analysis. Size distribution video data were acquired using the following settings: temperature: 25°C; frames: 60; duration: 2 s; cycles: 2; positions: 11; camera sensitivity: 80 and shutter value: 100. Data were analyzed using the ZetaView NTA software (version 8.05.12) with the following post acquisition settings: minimum brightness: 20; max area: 2000; min area: 5 and tracelength: 30.

### Proteomics.

Comparative proteomics was carried out on samples of BEVs produced in BHI and from BEVs and EVs isolated from the cecum of mice. Vesicles were isolated from 3 independent cultures for each condition. Each protein that was identified with the level of confidence determined by the FDR, was then further analyzed. Parental cells were from BHI cultures or the cecum of Bt colonized mice (3 replicates for each condition). Samples for proteomics analysis consisted of 50 μg of BEV or cell protein extract prepared and labeled at the Bristol University proteomics facility using TMT reagents (10-Plex format, Isobaric Mass Tagging kit, Thermo Scientific). Labeled samples were pooled and then fractionated using High pH Reverse Phase Liquid Chromatography. The resulting fractions were subjected to nano-LC MSMS using an Orbitrap Fusion Tribrid mass spectrometer with an SPS-MS3 acquisition method. Fragmentation of the isobaric tag released the low molecular mass reporter ions which were used to quantify the peptides. Protein quantitation was based on the median values of multiple peptides identified from the same protein, resulting in highly accurate protein quantitation between samples. The data sets were analyzed using the Proteome Discoverer v2.1 software and run against the Bt VPI-5482 or mouse database and filtered with a 5% (1%) FDR cutoff. The abundance values for each TMT channel were normalized so that all channels had the same total abundance.

### Proteomics data curation.

Raw results displayed a list of 3092 proteins identified in parent cells produced *in vitro* and *in vivo*. A hundred contaminant proteins (FALSE) were removed from the data. Using the 99% confidence level (<1% FDR), 213 additional proteins were removed. Proteins that were not found in BEVs (732) were also removed from the list resulting in a total of 2047 identified proteins (Table S2). For the abundance ratio of BEV proteins (mouse cecum versus BHI) those with a ratio ≥ 15 and a PSMs ≥ 10 were retained, resulting in a total of 113 proteins. To discriminate between proteins that are enriched in BEVs *in vivo*, the 51 proteins with an abundance ratio in the cell lysate (mouse cecum versus BHI) ≥ 3 were considered nonenriched whereas the 62 proteins with an abundance ratio in cell lysates (mouse cecum versus BHI) ≤ 3 were considered enriched in BEVs.

### Electron microscopy.

To visualize bacterial cells and the release of BEVs from the surface, cells were grown in BHI to early stationary phase and visualized by negative staining electron microscopy. 2 μL of liquid culture was applied to a 400-mesh gold TEM grid coated with Formvar/carbon. The sample was left to settle out for 5 min and 2 μL of 2× fixative (5% glutaraldehyde in 200 mM sodium cacodylate buffer, pH 7.2) was added and left for 5 min. The grid was then immersed for 10 min in 10 μL of 1× fixative, washed 5 times with 100 mM sodium cacodylate buffer, pH 7.2 and 5 times with ultrapure water (1 min each). The grid was air dried before negative staining in 2% aqueous Uranyl acetate-stain was applied and removed immediately. Grids were air dried and viewed in a Jeol 1230 TEM operated at an accelerating voltage of 80 kV. Images were recorded on a Gatan OneView 16MP digital camera. BEV suspensions were visualized using negative staining with TEM. Briefly, 4 μL BEV suspension was adsorbed to plasma-pretreated carbon-coated copper EM grids (EM Solutions) for 1 min before wicking off with filter paper and negatively staining with 1% Uranyl Acetate solution (BDH 10288) for 1 min. Grids were air-dried before analysis using a FEI Talos F200C electron microscope at 36,000×-92,×000 magnification with a Gatan OneView digital camera.

### BT_2086 (BSH) deletion mutant.

A 0.9 kb chromosomal DNA fragment upstream from *BT_2086* including the first 30 nucleotides of the 5′-end region was amplified by PCR using the primer pair BT2086-1 and -2 (Table 2). The product was cloned into the *Sac*I/*Bam*HI sites of the E. coli-*Bacteroides* suicide shuttle vector pGH014 (60). A 0.9 kb chromosomal DNA fragment downstream from *BT_2086*, including the last 40 nucleotides of the 3′-end region, was amplified by PCR using the primer pairs BT2086-3 and -4 (Table 2) and the fragment was cloned into the *Sal*I/*Pst*I sites of the pGH014-based plasmid already containing the corresponding upstream region. The resulting plasmid was mobilized from E. coli strain GC10 into Bt by triparental filter mating (60), using E. coli HB101(pRK2013) as the helper strain. Transconjugants were selected on BHIH agar containing gentamicin (200 mg/L) and tetracycline (1 mg/L). Determination of susceptibility to either tetracycline or erythromycin was done to identify recombinants that were tetracycline resistant and erythromycin susceptible after restreaking transconjugant bacteria on LB-agar containing tetracycline or both antibiotics. PCR analysis and sequencing were used to confirm allelic exchange. A transconjugant containing *ΔBT_2086::tetQ* was selected for further studies.

**TABLE 2:** Sequence of primers used in this study^*a*^

Primer	Sequence (5′→ 3′)
BT2086_1	AGTCGAGCTCGACGGACATCGTAGCTCCGT
BT2086_2	AGTCGGATCCCAATGCAACACCCGTAAGTT
BT2086_3	AGTCGTCGACAGGATAGTCAATCATTTACT
BT2086_4	AGTCCTGCAGTCGGGACACTTCGGAGGTGC
BT2757_1	GATAGAGGATCCCGAATATCAGAAAC
BT2757_2	CAGTCCGTGATTAGAATATTTGAATAAAGAGCAGTC
BT2757_3	CAAATATTCTAATCACGGACTGGAAACAAATTCAG
BT2757_4	GATGCCTGCCAGATATCCCCAG

a Restriction sites are underlined.

### BT_2757 (ASNase) deletion mutant.

*BT_2757* knockout strain was generated using the method developed by Garcia-Bayona and Comstock (61). Briefly, *BT_2757* flanking DNA fragments containing an overlap sequence were generated (upstream primers: BT2757-1 and -2, and downstream primers: BT2757-3 and -4, Table 2). Recombinant PCR was used to combine the fragments. BamHI and EcoRV sites were introduced during amplification flanking the fragment to allow for ligation into pLGB13 (61). E. coli PIR1^+^ (ThermoFisher Scientific) was used to carry out the cloning and as the donor strain. Bt VPI-5482 was conjugated using triple mating with E. coli HB101 (pRK2013) used as the helper strain (60). Transconjugants were selected on BHIS (BHIH supplemented with cysteine, 1 g/L and NaHCO_3,_ 2 g/L) agar containing gentamicin (200 μg/mL) and erythromycin (25 μg/mL) after 48 h. Two colonies were grown overnight in BHIH (with gentamicin (200 μg/mL) and erythromycin (25 μg/mL)) and streaked out on the counter selection plate of BHIH agar (gentamicin 200 μg/mL, anhydrotetracycline 100 ng/mL). Colonies lacking the gene were identified by colony PCR. Positive colonies were grown in BHIH (gentamicin (200 μg/mL)); anhydrotetracycline (100 ng/mL) and stored at 80°C in 10% glycerol in BHIH prior to use.

### Statistical analysis.

A principal-component analysis (PCA) was performed based on each protein normalized abundances using Metaboanalyst v5.0 online software (62). For enzyme assays, data were subjected to one-way ANOVA followed by Tukey’s multiple comparison *post hoc* test using GraphPad Prism 5 software. Statistically significant differences between two mean values were established by adjusted *P*-value < 0.05. Data are presented as the mean ± standard deviation.

### Bile salt hydrolase activity.

Thin layer chromatography to assess the activity and substrate specificity of BSHs in Bt were performed according to Sedláčková et al. 2015 (63). Bt strains were grown in 5 mL BHI for 16 h. The cultures were centrifuged at 9000 g for 10 min at 4°C, the cell pellets washed with 2 mL of PBS and resuspended in 5 mL of PBS. 500 μL of washed sample (or 5 × 10^9^ BEVs in 500 μL PBS) were mixed with 500 μL of substrate solution (Na-GCA 0.3% and Na-TCA 0.3% in PBS) and incubated for 16 h at 37°C. The TLC chamber containing the mobile phase (isoamyl acetate 40%, propionic acid 30% and 1-propanol 20% in water) was equilibrated for 30 min. The reaction mixtures were speed-vacuum dried, the pellets dissolved in 500 μL of methanol and the solution was centrifuged at 14 000 g at 4°C for 1 min. 3 μL of the supernatants and of the standards (cholic acid [CA], sodium taurocholic acid [Na-TCA] or sodium glycocholic acid [Na-GCA] 5 mM in methanol) were spotted onto silica gel plates (TLC Silica gel 60 F_254_, Merck). The plate was inserted into the chamber and allowed to run for about 40 min and removed when the solvent front was 1 to 2 cm from the top edges. The plate was dried at 110°C for 3 min and sprayed with a solution of phosphomolybdic acid (10% wt/vol in ethanol). The plate was dried again until spots were visible.

### Total protein extraction.

Cell pellets were resuspended in 250 μL of 0.2 M Tris-HCl (pH 7.2), and cell disruption via sonication was performed using eight 10-s pulses (amplitude, 6 μmol), with 30-s pauses on ice between each pulse. Cell extracts were obtained in the form of supernatants after centrifugation at 14,000 *g* for 30 min at 4°C.

### DPP-4 and asparaginase assays.

BEV and parent cells were prepared as described above except that the fractionation of the BEV samples was performed using a qEV/35 nm series size exclusion chromatography (SEC) column and the fractions were obtained according to manufacturer’s instructions (IZON Science, Lyon, France). BEVs typically elute in 6 fractions of 0.5 mL. DPP-4 assays were performed as described by Beauvais et al. (32). Briefly, 910 μL of 50 mM Tris HCl buffer (pH 7.5) and 50 μL Ala-Pro-pNA (5 mg/mL in methanol) were added to 40 μL of BEV suspension. To assess enzyme binding specificity, 0.33 μM the DPP-4 inhibitor vildagliptin (31) was added. The reaction mixtures were incubated at 37°C and the OD_405_ was measured at 1 min intervals for 100 min. The asparaginase assay was performed using the Asparaginase Activity assay kit (Abcam, ab107922) according to the manufacturer’s instructions. The amount of protein in BEVs was determined using the BCA protein assay kit for low concentrations (Abcam ab207002) according to manufacturer’s instructions. Protein values generally correlated with the concentration of particles determined by Zetaview (data not shown).

### Data availability.

The raw proteome data can be found in Table S2 in the supplemental material.
