# Supplementary material for: The Proteome of Extracellular Vesicles Produced by the Human Gut Bacteria Bacteroides thetaiotaomicron In Vivo Is Influenced by Environmental and Host-Derived Factors
Source: Appl Environ Microbiol. 2022 Aug 2;88(16):e00533-22. doi: 10.1128/aem.00533-22 (PMC9397113; doi:10.1128/aem.00533-22)
Supplement: Supplemental file 1 — Fig. S1 to S3. Download aem.00533-22-s0001.pdf, PDF file, 1.3 MB [file aem.00533-22-s0001.pdf]

Figure S1. Release of BEVs from the surface of Bt. The cells were grown in BHI media to early stationary phase and visualised by negative stain electron microscopy.

Figure S2. Volcano plots displaying ratios of protein abundances in vivo versus in vitro. The set thresholds were 0.05 for the p-value (n = 3) and 2 for the fold-change (FC). Features with > 50 % missing values were removed. Blue dots indicate protein that are significantly less abundant when obtained from in vivo conditions and red dots indicate proteins that are significantly more abundant.

Figure S3. Concentration and size of BEVs and EVs extracted from the mouse caeca. The concentration and size of particles was determined using ZetaView Nanoparticle Tracking Analyzer. The fractions were collected after SEC purifications of vesicle extracts from (A) EVs from germfree mice (GF) and germfree mice conventionalised with Bt WT (in vivo WT) (B) EVs from germfree mice (in vivo GF) and germfree mice conventionalised with Bt WT (in vivo WT) and BT_2757 deletion mutant (in vivo ASNase).

Figure S1

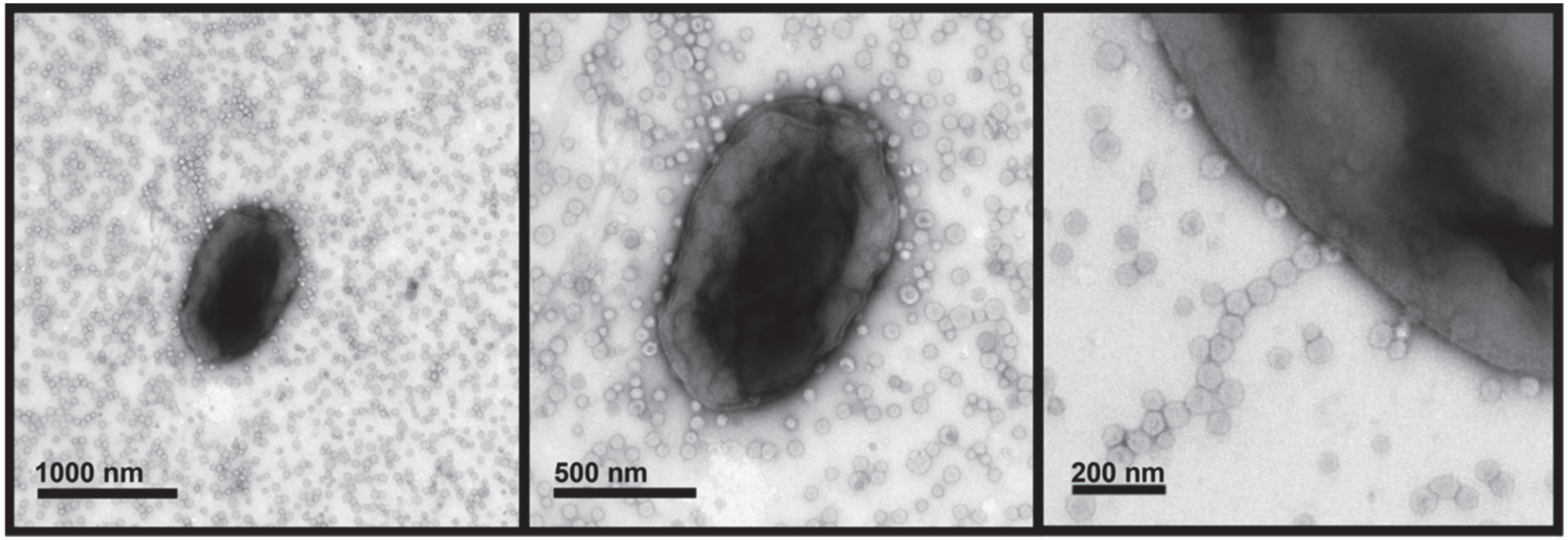

Figure S2

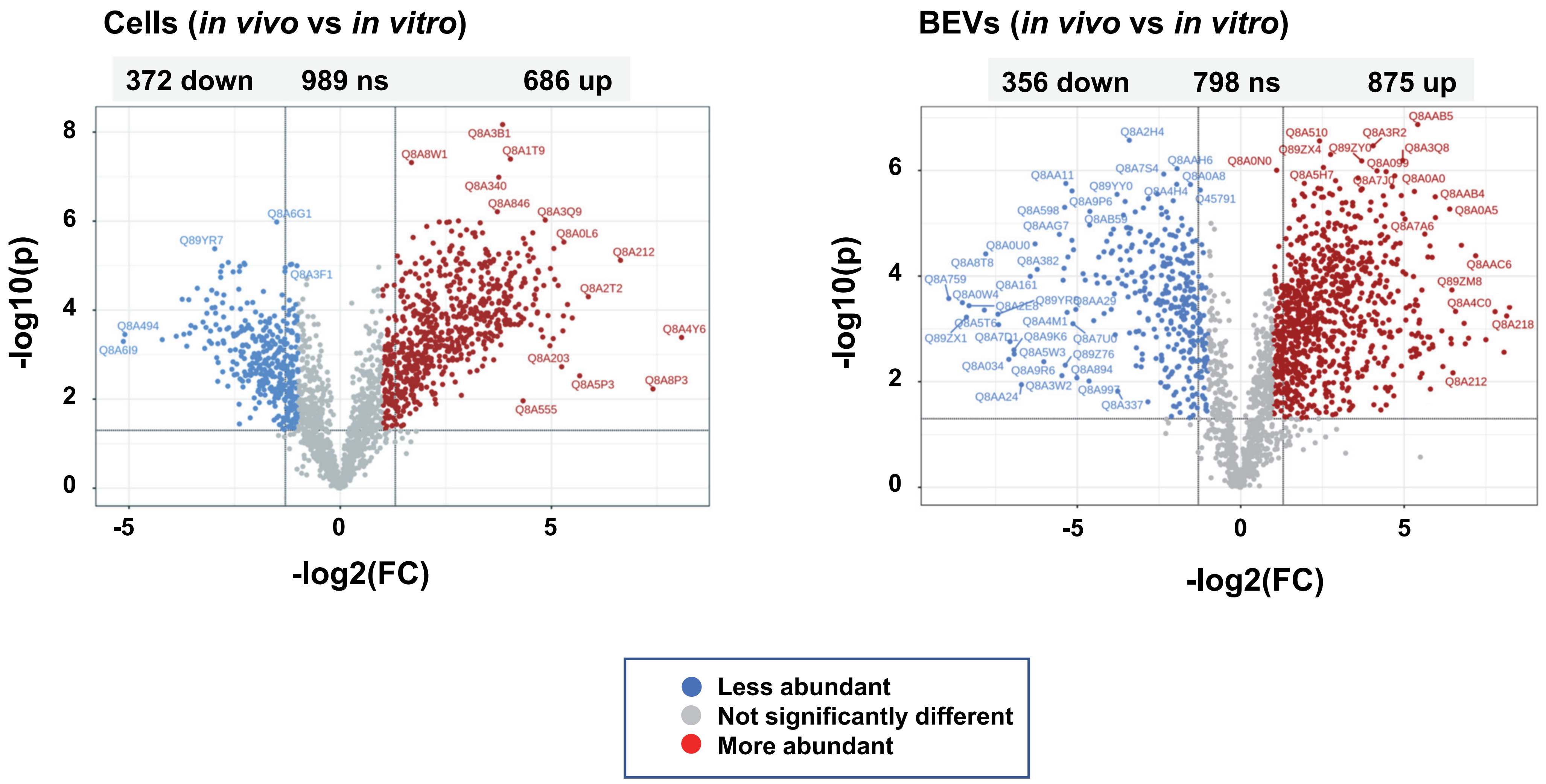

Cells (*in vivo* vs *in vitro*)
372 down
989 ns
686 up
-log10(p)
-log2(FC)
BEVs (*in vivo* vs *in vitro*)
356 down
798 ns
875 up
-log10(p)
-log2(FC)
Less abundant
Not significantly different
More abundant

Figure S3

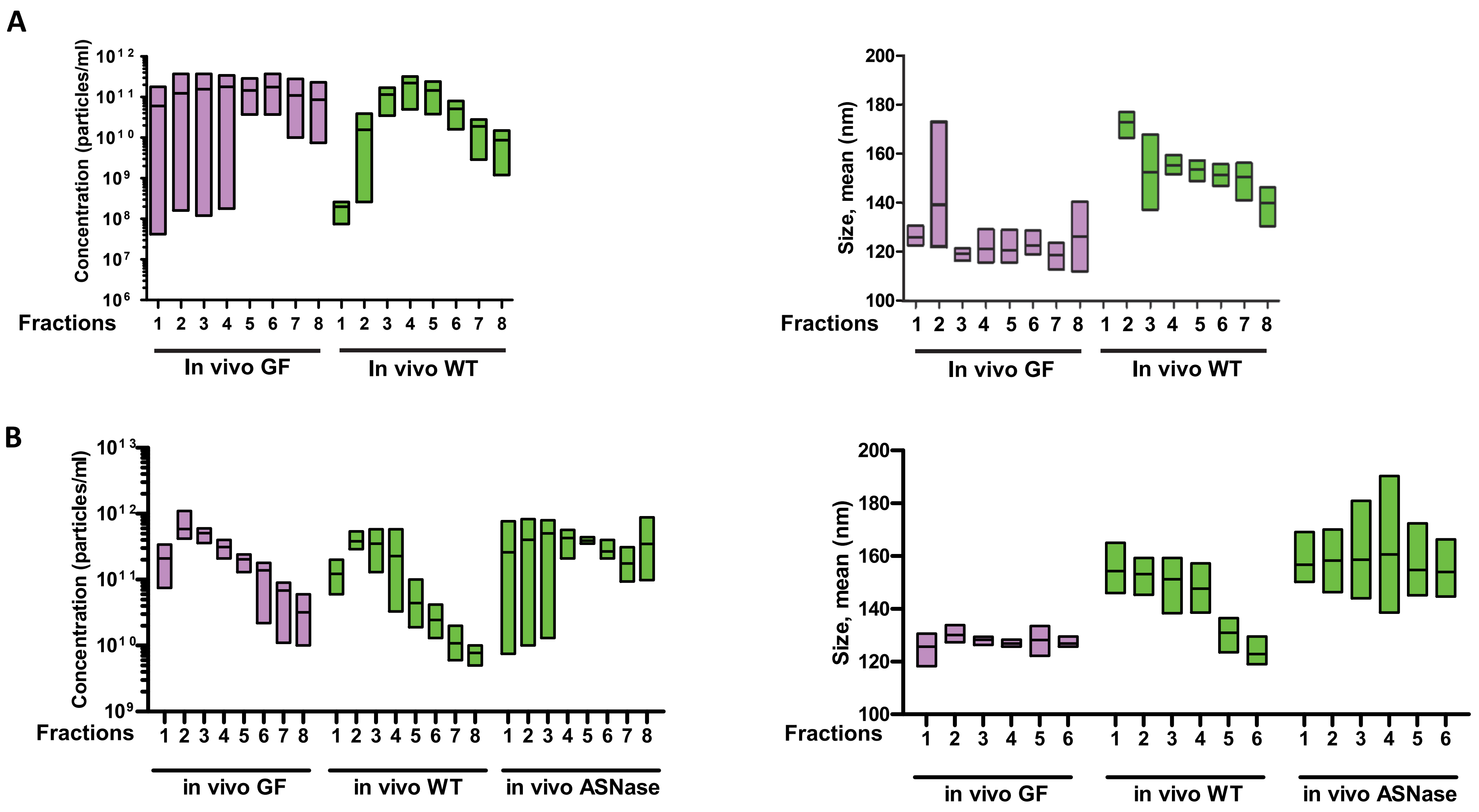
A
Concentration (particles/ml)
10^{12}
10^{11}
10^{10}
10^{9}
10^{8}
10^{7}
10^{6}
Fractions
1 2 3 4 5 6 7 8 1 2 3 4 5 6 7 8
In vivo GF
In vivo WT
Size, mean (nm)
200
180
160
140
120
100
Fractions
1 2 3 4 5 6 7 8 1 2 3 4 5 6 7 8
In vivo GF
In vivo WT
B
Concentration (particles/ml)
10^{13}
10^{12}
10^{11}
10^{10}
10^{9}
Fractions
1 2 3 4 5 6 7 8 1 2 3 4 5 6 7 8 1 2 3 4 5 6 7 8
in vivo GF
in vivo WT
in vivo ASNase
Size, mean (nm)
200
180
160
140
120
100
Fractions
1 2 3 4 5 6 1 2 3 4 5 6 1 2 3 4 5 6
in vivo GF
in vivo WT
in vivo ASNase
